# Effect of SARS-CoV-2 Breakthrough Infection on HIV Reservoirs and T-Cell Immune Recovery in 3-Dose Vaccinated People Living with HIV

**DOI:** 10.3390/v15122427

**Published:** 2023-12-14

**Authors:** Meng-Meng Qu, Bing Song, Bao-Peng Yang, Zerui Wang, Minrui Yu, Yi Zhang, Chao Zhang, Jin-Wen Song, Xing Fan, Ruonan Xu, Ji-Yuan Zhang, Chun-Bao Zhou, Fengxia Du, Fu-Sheng Wang, Hui-Huang Huang, Yan-Mei Jiao

**Affiliations:** 1 Department of Infectious Diseases, The Fifth Medical Center of Chinese PLA General Hospital, National Clinical Research Center for Infectious Diseases, Beijing 100039, China; qumm302@163.com (M.-M.Q.);; 2 Department of Gastroenterology, First Medical Center of Chinese PLA General Hospital, Beijing 100853, China; 3 Department of Pharmacy, Medical Supplies Center of PLA General Hospital, Beijing 100039, China

**Keywords:** breakthrough infection, HIV, HIV reservoirs, immune recovery, SARS-CoV-2, vaccine

## Abstract

People living with human immunodeficiency virus (PLWH) are a vulnerable population with a higher risk of severe coronavirus disease 2019 (COVID-19); therefore, vaccination is recommended as a priority. Data on viral reservoirs and immunologic outcomes for PLWH breakthrough infected with severe acute respiratory syndrome coronavirus 2 (SARS-CoV-2) are currently limited. In this study, we investigated the effects of SARS-CoV-2 breakthrough infection on hematological parameters, human immunodeficiency virus (HIV) reservoir size, and T-cell recovery in PLWH receiving antiretroviral therapy (ART) after SARS-CoV-2 booster vaccination. The results indicated that during breakthrough infection, booster vaccination with homologous and heterologous vaccines was safe in PLWH after receiving two doses of inactivated vaccination. The absolute CD4 counts decreased in the heterologous group, whereas the CD8 counts decreased in the homologous booster group after breakthrough infection in PLWH. Breakthrough infection increased HIV reservoirs and was associated with increased T-cell activation in PLWH who received virally suppressed ART and a 3-dose vaccination. According to our data, the breakthrough infection of SARS-CoV-2 may put PLWH at a greater risk for increased HIV reservoirs, even if these individuals were virally suppressed with ART after 3-dose SARS-CoV-2 vaccination.

## 1. Introduction

The coronavirus disease 2019 (COVID-19) pandemic caused by severe acute respiratory syndrome coronavirus 2 (SARS-CoV-2) has threatened a heavy burden on global health and society’s economy. Despite controversies, people living with human immunodeficiency virus (PLWH) are a highly vulnerable population with an increased risk of mortality despite the beneficial effects of antiretroviral therapy (ART) [1,2]. Therefore, vaccination against SARS-CoV-2 is recommended for PLWH. An earlier report demonstrated an increased risk of COVID-19 morbidity and mortality in the immunodeficient population, accompanied by poor neutralizing antibody responses, compared with human immunodeficiency virus (HIV)-negative individuals [3]. The poor serological response to vaccines in PLWH is in line with other vaccines, such as those for influenza or hepatitis B virus [4,5]. Inconsistently, several studies have suggested that a comparable safety, humoral and T-cell immune response against SARS-CoV-2 is elicited in PLWH with higher CD4 counts and HIV-negative populations [6,7,8]. Recent observational studies have indicated a higher incidence of breakthrough infection in PLWH than in HIV-negative populations, even after booster vaccination [9,10,11]. However, data on the safety and immunogenicity during breakthrough infection in PLWH with inactivated SARS-CoV-2 vaccine are lacking. 

Persistence of a stable latent HIV reservoir in resting CD4^+^ T cells is the main obstacle to HIV eradication. Previous studies have shown that standard vaccination against common pathogens can increase HIV reservoirs in the context of successful ART [12]. It has been reported that COVID-19 vaccination positively affects CD4 counts and viral markers in PLWH, as indicated by increased CD4 counts after vaccination and an increased percentage of patients with HIV-RNA < 50 copies/mL after the second and third vaccinations [13]. Another report demonstrated no significant changes in the HIV reservoir size after SARS-CoV-2 booster vaccination [14]. Owing to the limited number of studies, it remains uncertain whether COVID-19 vaccines can affect HIV reservoirs. Furthermore, given the widespread and possible long-term existence of SARS-CoV-2, it is important to investigate whether breakthrough infection modulates HIV reservoirs in PLWH receiving ART.

Here, we studied the effects of SARS-CoV-2 breakthrough infection on clinical characteristics, CD4 counts, HIV reservoir size, and T-cell immune recovery in a cohort of PLWH after booster vaccination, followed by a 2-dose inactivated vaccine. 

## 2. Materials and Methods

### 2.1. Human Subjects and Study Design

As part of the vaccination program for special populations, a cohort of HIV-infected patients was enrolled from the Fifth Medical Center of Chinese PLA General Hospital from August 31, 2021, onwards. The inclusion criteria were as follows: age 18–60 years; receiving ART with plasma HIV RNA < 20 copies/mL; and no history of SARS-CoV-2 infection. The exclusion criteria were as follows: history of anaphylactic response to vaccine components; current opportunistic infection; Hepatitis B virus, Hepatitis C virus, or influenza virus infection; and tumor and autoimmune diseases. 

**Figure 1:**
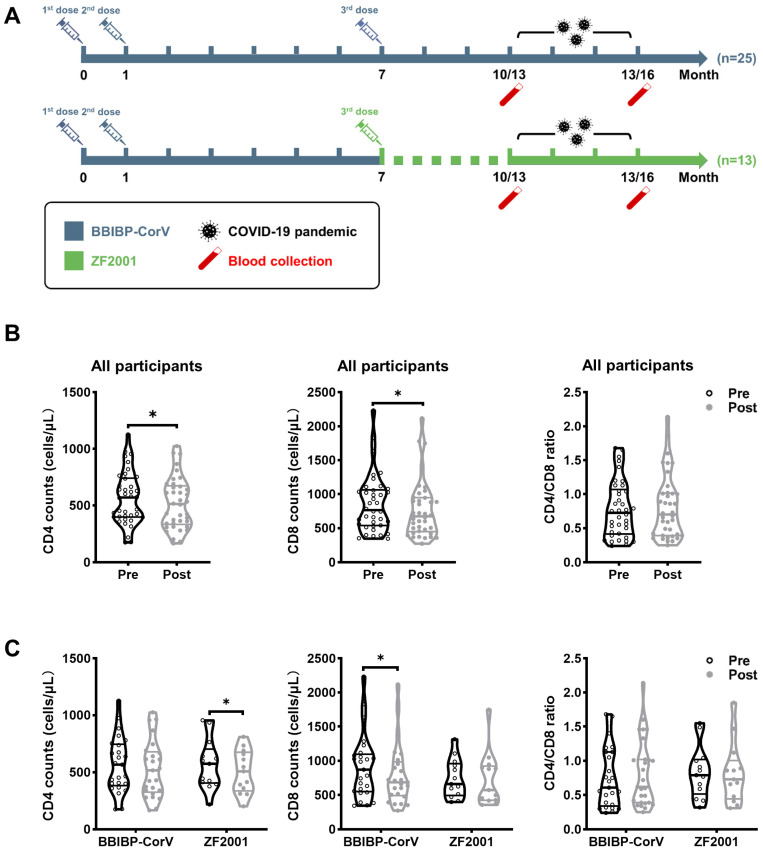
Study design and dynamics of CD4 counts, CD8 counts, and CD4/CD8 ratio during SARS-CoV-2 breakthrough infection. (**A**) The schematic diagram described the vaccination strategy in which the first two doses of BBIBP-CorV were injected intramuscularly with a 28-day interval and the homologous booster BBIBP-CorV or heterologous booster ZF2001 was administered 6 months after the second dose. The COVID-19 pandemic occurred 10 or 13 months after the first dose. Blood samples were collected in the 10th/13th and 13th/16th month. Comparisons of CD4 counts, CD8 counts, and CD4/CD8 ratio within all participants (**B**) and within the BBIBP-CorV homologous booster and ZF2001 heterologous booster groups (**C**) were made between pre- and post-breakthrough infection. Data were analyzed using the Mann–Whitney U test and paired *t*-test. * *p* < 0.05.

As shown in Figure 1A, all participants received a 2-dose series of inactivated Sinopharm COVID-19 vaccine (BBIBP-CorV) with a 28-day interval. These individuals received a third dose of BBIBP-CorV or Zifivax COVID-19 recombinant protein subunit vaccine (ZF2001) at a 6-month interval with the second dose. Peripheral blood samples were collected from all participants at the follow-up. Detailed demographic, epidemiological, clinical, and laboratory characteristics were collected from the hospital’s electronic medical record system.

While the COVID-19 pandemic (with Delta and Omicron variants predominant) occurred at the end of 2022, we tested the SARS-CoV-2 among the HIV population. The SARS-CoV-2 breakthrough infection was confirmed by nucleic acid or antigen testing. We chose the positive population to analyze the samples before (pre-, 10th or 13th month of follow-up) and after (post-, 13th or 16th month of follow-up) breakthrough infection. 

### 2.2. Sample Preparation

Whole blood was collected in ethylenediaminetetraacetic acid-containing tubes (BD Biosciences, San Jose, CA, USA) and processed within 12 h from the blood draw. Peripheral blood mononuclear cells (PBMCs) were isolated from the blood by Ficoll–Hypaque density gradient centrifugation and stored in liquid nitrogen until use.

### 2.3. Flow Cytometry Analysis

The expression of markers of T-cell phenotype and activation was assessed using cryopreserved PBMCs. Cells were thawed, washed, and stained in the dark with Fixable Viability Stain 700 and the following fluorescent-conjugated cell surface marker antibodies: CD3-BUV737 (clone SK7), CD4-BUV496 (clone SK3), CD8-PE-Cy7 (clone RPA-T8), CD45RA-FITC (clone HI100), CD27-BV421 (clone M-T271), CCR7-PE (clone 3D12), CD38-BV605 (clone HB7), and HLA-DR-BV711 (clone G46-6). Samples were acquired using a BD FACSymphony^TM^ A5 flow cytometer, and the data were analyzed using FlowJo version 10.7.1. 

### 2.4. Quantification of HIV-1 DNA and RNA

Total cellular DNA and RNA were extracted from PBMCs using the Qiagen QIAsymphony DNA Mini Kit (Qiagen, Valencia, CA, USA) and HiPure Total RNA Plus Mini Kit (Magen, Guangzhou, China), respectively. A fluorescence-based real-time SUPBIO HIV Quantitative Detection Kit (SUPBIO, Guangzhou, China) was used to amplify and quantify the HIV DNA and RNA. The DNA and RNA copy numbers were normalized to 1 × 10^6^ PBMCs.

### 2.5. Statistical Analysis

GraphPad Prism version 8.0 software (GraphPad, Inc., San Diego, CA, USA) was used to analyze the data. Continuous variables are expressed as the median interquartile range, and categorical variables are expressed as counts (%). The Pearson chi-square test was used to compare categorical data between groups. The Mann–Whitney U test was used to compare two groups, and the paired *t*-test was used to compare the changes within the group. Correlations between two continuous variables were identified using the Spearman rank correlation test. *p* values < 0.05 were considered to indicate that the results were statistically significant.

## 3. Results

### 3.1. Characteristics of Study Participants

To investigate the effect of SARS-CoV-2 breakthrough infection on HIV-infected individuals, 38 individuals were included in this study and classified according to the booster vaccination: BBIBP-CorV = 25, ZF2001 = 13 (Figure 1A). The characteristics of the participants are summarized in Table 1. Participants who received the BBIBP-CorV booster consisted of 24 males (96.0%) and 1 female (4.0%), with a median age of 39 years (IQR 30–49). In the ZF2001-vaccinated cohort, the median age was 41 years (IQR 37–56), and 12 participants (92.3%) were males. The interval between the last vaccination and breakthrough infection was either 3 months (BBIBP-CorV = 32.0%; ZF2001 = 23.1%) or 6 months (BBIBP-CorV = 68.0%; ZF2001 = 76.9%).

There were no significant differences in the baseline CD4 and CD8 counts, CD4/CD8 ratio, ART time, or ART regimen. All individuals had undetectable plasma viremia (<20 copies/mL) at the time of enrollment.

### 3.2. Hematological Parameters after Breakthrough Infection in PLWH

No deaths occurred during the follow-up visit and no severe illness or serious adverse events occurred among the participants. We also investigated the effects of breakthrough infection on hematological variables in the two groups (Table 2). In the homologous BBIBP-CorV group, there was a significant decrease in hemoglobin (153 vs. 155, *p* = 0.025) and creatinine (85 vs. 87, *p* = 0.035) levels after breakthrough infection, although they did not exceed the normal range. During the entire process of breakthrough infection, almost all other hematological parameters fluctuated within the normal range without any significant differences. Collectively, these results demonstrated that vaccination with homologous BBIBP-CorV booster or heterologous ZF2001 booster appears safe in PLWH who had previously received two doses of inactivated BBIBP-CorV.

### 3.3. Decreased CD4 and CD8 Counts after Breakthrough Infection in PLWH

Next, we assessed whether the CD4 counts, CD8 counts, and CD4/CD8 ratio were altered by breakthrough infection in the study participants. As shown in Figure 1B, both the CD4 and CD8 counts after breakthrough infection were significantly lower than those before infection, whereas the CD4/CD8 ratio remained similar between the groups. Moreover, the heterologous ZF2001 group displayed significantly lower levels of CD4 counts but similar CD8 counts after breakthrough infection (Figure 1B). Conversely, the homologous BBIBP-CorV booster group showed the same levels of CD4 counts but substantially lower CD8 counts after breakthrough infection. The CD4/CD8 ratio remained unchanged even after breakthrough infection in both booster groups. Thus, breakthrough infection caused an obvious decrease in CD4 counts in heterologous booster individuals and a decrease in CD8 counts for the homologous booster group in PLWH.

### 3.4. Changes of HIV Reservoir Size after Breakthrough Infection in PLWH

**Figure 2:**
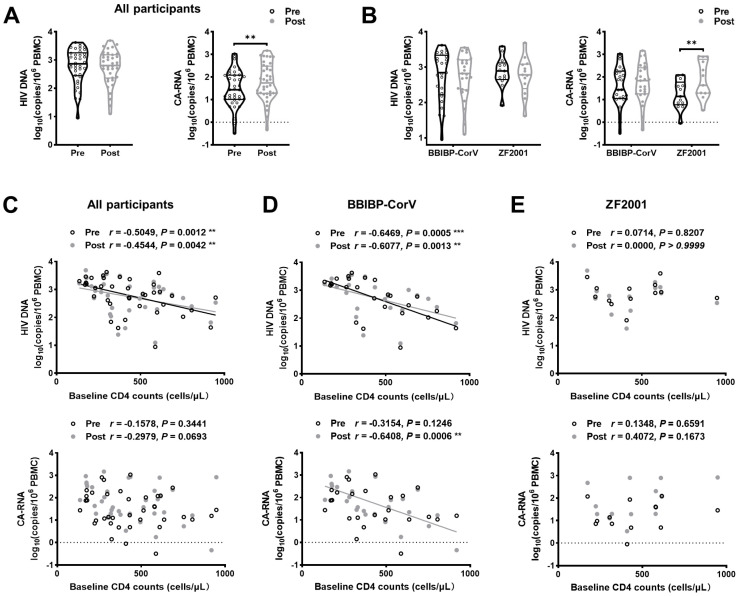
Dynamics of HIV reservoirs. The dynamics of HIV DNA and CA-RNA during breakthrough infection are shown in (**A**), and changes in HIV DNA and CA-RNA levels in the BBIBP-CorV homologous booster and ZF2001 heterologous booster groups are presented in (**B**). Correlation between baseline CD4 counts and HIV DNA and CA-RNA levels are depicted in (**C**) all participants, (**D**) BBIBP-CorV homologous booster group, and (**E**) ZF2001 heterologous booster group pre- and post-breakthrough infection, respectively. (The black hollow points and gray solid points indicate pre- and post-breakthrough infections, respectively). Data were analyzed using Mann–Whitney U test and paired *t*-test. ** *p* < 0.005, *** *p* < 0.001.

Previous studies have suggested that influenza/hepatitis B vaccination is associated with an increase in HIV reservoirs [12,15]. Few studies have focused on HIV reservoirs after vaccination and breakthrough infection. Recently, a brief report demonstrated that there were no significant differences in the levels of HIV reservoirs after SARS-CoV-2 booster vaccination [14]. Therefore, we evaluated the effect of breakthrough infection on the HIV reservoir parameters in PLWH who had received a booster vaccination after two doses of BBIBP-CorV. As shown in Figure 2A, no significant differences in the level of HIV DNA but a significant increase in the level of HIV cell-associated RNA (CA-RNA) were found after breakthrough infection. The heterologous group showed higher HIV CA-RNA levels after breakthrough infection (Figure 2B).

Next, we analyzed whether the HIV reservoir parameters before and after breakthrough infection were correlated with baseline CD4 counts. Higher baseline CD4 counts were associated with lower HIV DNA levels before (*r* = −0.5049, *p* = 0.0012) and after breakthrough infection (*r* = −0.4544, *p* = 0.0042; Figure 2C). However, there was no correlation between CD4 counts and HIV CA-RNA levels before or after breakthrough infection (Figure 2C). For the homologous boosters, higher HIV DNA levels pre-infection were strongly negatively correlated with lower baseline CD4 counts (*r* = −0.6469, *p* = 0.0005) rather than HIV CA-RNA (*r* = −0.3154, *p* = 0.1246; Figure 2D). After breakthrough infection, baseline CD4 counts were negatively correlated with HIV DNA and CA-RNA levels, respectively (*r* = −0.6077, *p* = 0.0013; *r* = −0.6408, *p* = 0.0006; Figure 2D). In the heterologous group, there was no correlation between CD4 counts and HIV DNA levels before and after breakthrough infection, whereas baseline CD4 counts were negatively associated with HIV CA-RNA post-breakthrough infection (*r* = −0.4072, *p* = 0.1673). Overall, these data demonstrate a trend of increasing HIV reservoirs in PLWH after breakthrough infection, especially in the heterologous booster individuals. 

### 3.5. T-Cell Subset Alterations before and after Breakthrough Infection in PLWH

**Figure 3:**
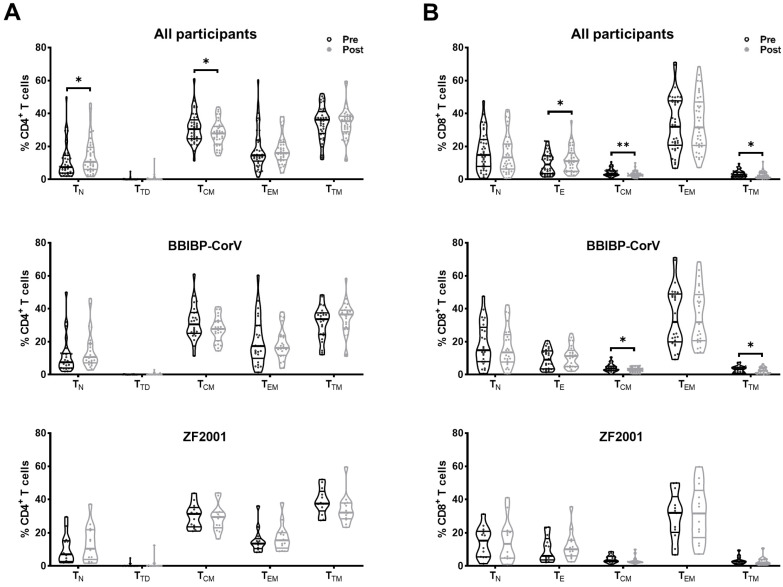
Changes of T-cell subsets during breakthrough infection. According to the expression of canonical markers, T-cell subsets were defined as follows: naïve (T_N_, CD45RA^+^CD27^+^CCR7^+^), central memory (T_CM_, CD45RA^−^CD27^+^CCR7^+^), transitional memory (T_TM_, CD45RA^−^CD27^+^CCR7^−^), effector memory (T_EM_, CD45RA^−^CD27^−^CCR7^−^), and terminal differentiated/effector (T_TD_/T_E_, CD45RA^+^CD27^+^CCR7^−^). The percentages of (**A**) CD4^+^ T-cell subsets and (**B**) CD8^+^ T-cell subsets were compared during breakthrough infection. Data were analyzed using Mann–Whitney U test. * *p* < 0.05, ** *p* < 0.005.

To determine the changes of CD4^+^ and CD8^+^ T-cell subset composition among PLWH during breakthrough infection, we used flow cytometry to analyze T-cell subsets according to the expression of CD45RA, CD27, and CCR7 (Figure S1). In CD4^+^ T cells, the frequency of naïve (T_N_) cells increased, but the frequency of central memory (T_CM_) cells decreased in PLWH post-breakthrough infection compared to pre-breakthrough infection (Figure 3A). Similar changes in T_N_ and T_CM_ cell frequencies, without significant differences, were observed in both homologous and heterologous booster groups. The frequency of terminally differentiated (T_TD_) cells increased in the BBIBP-CorV group but decreased in the ZF2001 group. In CD8^+^ T cells, the frequency of effector (T_E_) cells increased, whereas the frequencies of T_CM_ and transitional memory (T_TM_) cells decreased after breakthrough infection (Figure 3B). Both homologous and heterologous booster individuals showed similar alterations in CD8^+^ T-cell subset composition (Figure 3B).

### 3.6. Cell Activation after Breakthrough Infection in PLWH

**Figure 4:**
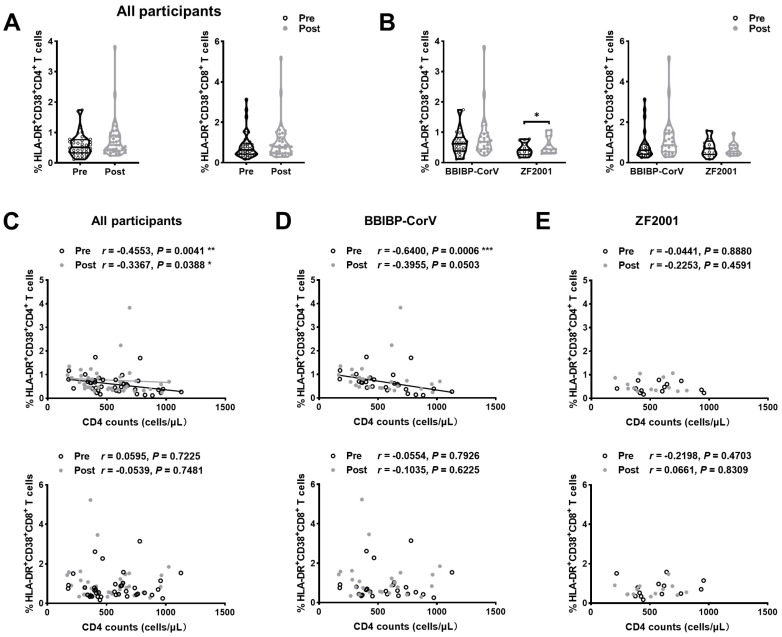
Changes in T-cell activation. During breakthrough infection, the dynamics of T-cell activation are pictured in (**A**) and the changes in CD4^+^ and CD8^+^ T-cell activation in the BBIBP-CorV homologous booster and the ZF2001 heterologous booster group are displayed in (**B**). Correlation between baseline CD4 counts and activation of CD4^+^ T and CD8^+^ T cells are calculated for (**C**) all participants, (**D**) BBIBP-CorV homologous booster group, and (**E**) ZF2001 heterologous booster group before and after breakthrough infection, respectively. Data were analyzed using Mann–Whitney U test and paired *t*-test. * *p* < 0.05, ** *p* < 0.005, *** *p* < 0.001.

To further examine the impact of breakthrough infection on T cells, the expression of activation markers (HLA-DR and CD38) on T cells was analyzed. We found that the frequencies of HLA-DR^+^CD38^+^CD4^+^ and HLA-DR^+^CD38^+^CD8^+^ T cells were higher in PLWH post-breakthrough infection than in those who were pre-infected (Figure 4A). The frequency of HLA-DR^+^CD38^+^CD4^+^ T cells significantly increased after breakthrough infection compared to that before infection in heterologous booster individuals (Figure 4B). Next, correlation analyses were performed between T-cell activation and CD4 counts in PLWH before and after breakthrough infection. A moderate negative correlation between CD4^+^ T-cell activation and CD4 counts was observed pre- and post-infection, respectively (*r* = −0.4553, *p* = 0.0041; *r* = −0.3367, *p* = 0.0388; Figure 4C), which was confirmed as significant in the BBIBP-CorV booster group (*r* = −0.6400, *p* = 0.0006; *r* = −0.3955, *p* = 0.0503; Figure 4D) but only marginal in the heterologous ZF2001 group (*r* = −0.0441, *p* = 0.8880; *r* = −0.2253, *p* = 0.4591; Figure 4E). On the contrary, no correlation was observed between CD4 counts and CD8^+^ T-cell activation before and after breakthrough infection, respectively (*r* = −0.0595, *p* = 0.7225; *r* = −0.0539, *p* = 0.7481; Figure 4C). There was no correlation between CD4 counts and HLA-DR^+^CD38^+^CD8^+^ T-cell frequency in the BBIBP-CorV and ZF2001 booster groups (Figure 4D,E). 

**Figure 5:**
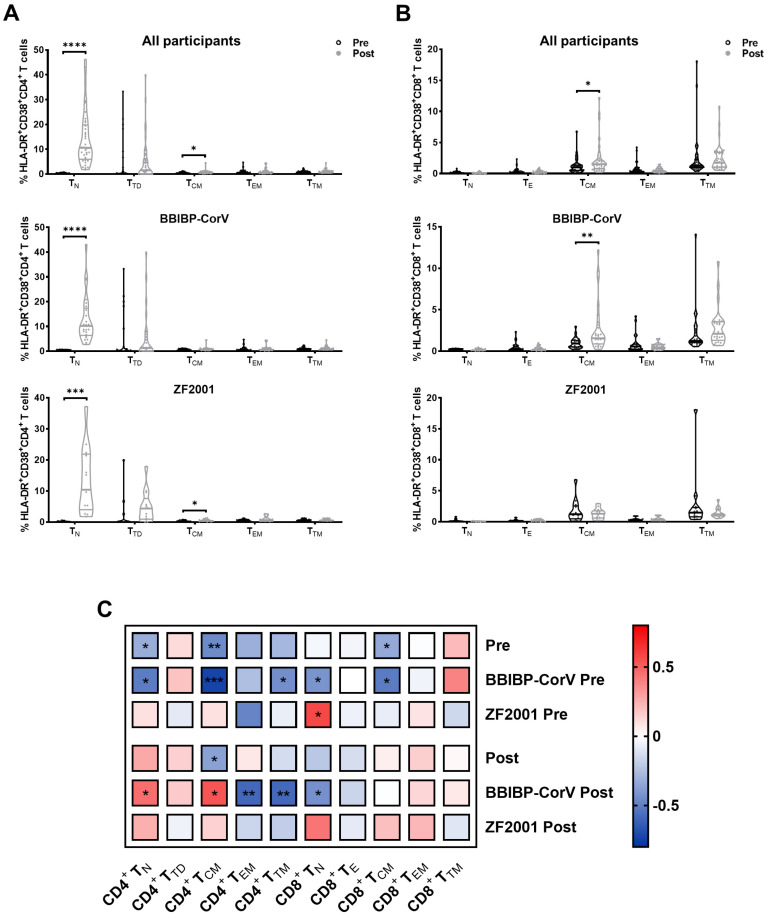
Dynamics of T-cell subset activation and its association with T-cell recovery during breakthrough infection. (Comparisons of the activation for each CD4^+^ T-cell subset (**A**) and CD8^+^ T-cell subset (**B**) are shown during breakthrough infection. Correlation between current CD4 counts and levels of T-cell subset activation in all participants, BBIBP-CorV homologous booster group, and ZF2001 heterologous booster group are calculated (**C**) before and after breakthrough infection, respectively. Data were analyzed using Mann–Whitney U test. * *p* < 0.05, ** *p* < 0.005, *** *p* < 0.001, **** *p* < 0.0001.

We then examined the activation of the T-cell subset and their association with CD4 counts. Strikingly, PLWH had a significant increase in the activation of CD4^+^ T_N_, CD4^+^ T_CM_, and CD8^+^ T_CM_ after the breakthrough infection (Figure 5A,B). Compared to the pre-infection timepoint, a significant increase in the activation of CD4^+^ T_N_ and CD8^+^ T_CM_ was found in the BBIBP-CorV individuals, whereas higher CD4^+^ T_N_ activation was observed in the ZF2001 booster group. Figure 5C showed the association between the activation of T-cell subsets and CD4 counts. The frequencies of HLA-DR^+^CD38^+^ T cells of CD4^+^ T_N_, CD4^+^.

T_CM_, CD4^+^ T_TM_, CD8^+^ T_N_, and CD8^+^ T_CM_ were found to have a significant negative correlation with the pre-infection CD4 counts in the BBIBP-CorV group. In addition, we found a significant negative association with CD4^+^ T_EM_, CD4^+^ T_TM_, and CD8^+^ T_N_ activation but a positive association with CD4^+^ T_N_ and CD4^+^ T_CM_ activation post-infection in the BBIBP-CorV group.

Collectively, these data demonstrated that CD4^+^ T-cell activation was significantly increased during breakthrough infection in the ZF2001 booster group, in line with the obvious increase in HIV reservoir size and decreased CD4 counts.

## 4. Discussion

PLWH are more susceptible to the severe effects of COVID-19 than others, owing to their immunosuppressed state and interruptions in HIV treatment and care [16,17]. As PLWH also have a compromised immune system, it is crucial to implement effective immune strategies to ensure their protection. However, for this particular population, the introduction of foreign antigens may result in unpredictable consequences. Reports have suggested that COVID-19 vaccination may have negative effects on PLWH, including HIV reservoir rebound and decreased CD4 counts [18,19]. Various studies have evaluated the safety and effectiveness of different vaccination strategies on PLWH, mainly focusing on COVID-19 mRNA vaccines given in single or multiple doses [20,21,22,23]. A recent study showed that mRNA booster vaccination may lead to HIV reservoirs rebound in elderly PLWH with unsuppressed viremia but is safe for ordinary elderly PLWH [24]. However, our understanding of the impact of other heterologous sequential vaccination strategies against COVID-19 in PLWH remains limited. In addition, studying the impact of breakthrough infection on PLWH is particularly important during the intense ongoing Omicron transmission period.

Here, we provided evidence of the safety of timely immunization with SARS-CoV-2 booster vaccines in PLWH. Moreover, there were also no deaths or severe cases in our cohort during the COVID-19 pandemic at the end of 2022, to some extent indicating the protective effectiveness of our vaccination strategy for populations with a risk of immune status such as PLWH. Several studies have been conducted on vaccine hesitancy in PLWH [25,26]. Higher COVID-19 vaccine hesitancy was associated with older age, lower educational level, chronic diseases, lower CD4 counts, and psychological factors, such as severe anxiety and depression. Research on the effectiveness and safety of COVID-19 vaccines for PLWH, including this study, will provide evidence to support vaccination behavior among individuals willing to vaccinate in the future. 

Currently, there are limited data available regarding the impact of infection and vaccination on viral suppression in PLWH. The earliest study suggested that COVID-19 infection does not affect HIV virological parameters [27]. In this study, we observed a significant decline in CD4 counts and a partially increased HIV reservoir after breakthrough infection. These results were consistent with those of a previous report showing a statistically insignificant decline in CD4 counts and a trend of increasing HIV-1 viral load after COVID-19 recovery in PLWH co-infected with SARS-CoV-2 [28]. However, we also noticed that both homologous and heterologous vaccination strategies could curb the expansion of the HIV reservoir in PLWH, particularly the increase of HIV DNA. HIV DNA includes replication-competent and defective proviruses, whereas CA-RNA, which consists of distinguishingly spliced transcripts produced by HIV, can be considered a perfect marker of the active reservoir [29,30]. The existence of a latent reservoir is the reason why HIV cannot be completely eliminated despite receiving long-term ART and successful suppression of viral replication [31]. The increased HIV reservoir after breakthrough infection may be accompanied by the activation of the viral reservoir, especially in patients with severe or critical COVID-19. In addition, with the continuous occurrence of new SARS-CoV-2 sub-variants, PLWH are still at high risk of being re-infected in the future, which may have a greater impact on the viral reservoir. We hope that the dangerous situation in PLWH can be reversed by administering the COVID-19 vaccine to them.

As more SARS-CoV-2 variants emerge that increasingly escape neutralizing antibody responses, CD4^+^ and CD8^+^ T-cell responses are likely to be important immunological mediators that contribute to viral control and clearance [32]. Previous studies have suggested that a rapid and extensive recall of T cells occurs after breakthrough infection, which benefits virological control [33]. Data regarding the differences in T-cell responses after breakthrough infection between the homologous and heterologous booster groups are still controversial [34,35]. Data from our study demonstrated that the heterologous vaccine regime induced a significant increase in CD4^+^ T-cell activation compared to that in homologous booster individuals. Broad T-cell immune activation was observed in patients enrolled in our study. The notable increase in the frequency of T_N_ may help maintain the size and functionality of the patient’s CD4^+^ T-cell pool following breakthrough infection. Importantly, this effect can even be achieved in patients with lower baseline CD4^+^ T-cell levels using allogeneic immunity. In contrast, the enhancement of T-cell immunity through the homologous strategy has limitations. It also resulted in the expansion of the naïve CD4^+^ Tcell pool in breakthrough-infected individuals; however, the frequency of activation did not show a significant increase. 

Under the impact of heterologous vaccination strategies, the tendency of CD8^+^ T cells to differentiate into memory cell types is more noticeable. Although the heterologous strategy cannot increase the overall frequency and level of activation of CD8^+^ T cells in patients with breakthrough infection, it may still decrease the patient’s viral load by expanding the memory and effector pool of CD8^+^ T cells. Furthermore, the activation of CD4^+^ T cells was negatively correlated with CD4 counts before and after breakthrough infection, particularly in heterologous booster individuals. The mechanisms underlying the changes in T cells warrant further investigation. 

Our study has several limitations. Firstly, there was a lack of PLWH without vaccination against SARS-CoV-2, which is also an important control to compare the immunological response to SARS-CoV-2 breakthrough infection with or without complete vaccination. At present, sporadic clinical case studies show that the systemic inflammation caused by COVID-19 will reactivate the potential HIV reservoir and temporarily increase the viral load. More comprehensive cohort studies are still needed to provide more evidence. Secondly, the viral reservoir size was over-evaluated because this technology includes not only intact provirus but also defective provirus. Thirdly, the cohorts included in this study were relatively small, which limits the generalizability of the results. The results may need to be confirmed in expanded cohort studies. Finally, some researchers have indicated that both inactivated and recombinant protein vaccines can influence virus-specific T-cell immunity [36,37]. However, we did not specifically investigate this aspect in our study. By evaluating the virus-specific T-cell immunity, more specific and focused conclusions may be drawn.

Despite these limitations, our study confirmed that the sequential COVID-19 vaccination strategy is safe and provided protection to PLWH during the pandemic. In addition, this study investigated, for the first time, the alteration of HIV reservoirs and their association with T-cell recovery during SARS-CoV-2 breakthrough infection in HIV-infected individuals. To avoid HIV reservoir reactivation, a heterologous booster immunization approach may be more beneficial than the homologous strategy. Altogether, our results provide important evidence for vaccination strategies among the immunodeficient population, which may benefit future global vaccination plans.

## Figures and Tables

**Table 1:** Baseline characteristics of enrolled participants in this study.

	BBIBP-CorV (n = 25)	ZF2001 (n = 13)	*p*_Value
Sex (%)			0.629
Male	24 (96.0%)	12 (92.3%)	
Female	1 (4%)	1 (7.6%)	
Age (years)	39 (30–49)	41 (37–56)	0.185
SARS-CoV-2 vaccine			
1st dose	BBIBP-CorV	BBIBP-CorV	
2nd dose	BBIBP-CorV	BBIBP-CorV	
3rd dose	BBIBP-CorV	ZF2001	
Pre-breakthrough infection (%)			0.565
month 10	8 (32.0%)	3 (23.1%)	
month 13	17 (68.0%)	10 (76.9%)	
CD4 counts (cells/µL)	377 (278–588)	426 (305–580)	0.808
CD8 counts (cells/µL)	526 (474–723)	565 (436–703)	0.761
CD4/CD8 ratio	0.64 (0.35–1.04)	0.73 (0.58–0.89)	0.416
Viral load (copies/mL)	<20	<20	-
ART time (years)	4 (3–5)	6 (4–7)	0.213
ART regimen (%)			0.395
3TC + TDF + LPV/r	2 (8%)	1 (8%)	
3TC + TDF + EFV	21 (84%)	9 (69%)	
3TC + AZT + NVP	2 (8%)	1 (8%)	
3TC + AZT + EFV	0	1 (8%)	
E/C/F/TAF	0	1 (8%)	

All indicators except gender are shown as medians (interquartile range). BBIBP-CorV, Sinopharm COVID-19 vaccines (Covilo, inactivated vaccine); ZF2001, Zifivax COVID-19 vaccine (recombinant protein subunit vaccine); ART, antiretroviral therapy; 3TC, lamivudine; TDF, tenofovirdisoproxil; LPV/r, ritonavir-boosted lopinavir; EFV, efavirenz; AZT, azidothymidine; DTG, dolutegravir; E/C/F/TAF, elvitegravir, cobicistat, emtricitabine, and tenofovir alafenamide.

**Table 2:** Hematology characteristics of study participants before and after breakthrough infection.

	BBIBP-CorV (n = 25)			ZF2001 (n = 13)		
	Pre	Post	*p*_Value	Pre	Post	*p*_Value
WBC (109/L)	5.78 (5.36–6.61)	6.01 (4.92–7.45)	0.380	4.06 (3.95–5.58)	4.80 (3.85–5.77)	0.339
HGB (g/L)	155 (147–163)	153 (146–161)	0.025 *	155 (143–162)	151 (146–162)	0.537
PLT (109/L)	250 (206–300)	242 (217–287)	0.864	218 (197–255)	230 (202–253)	0.201
CRE (µmol/L)	87 (82–91)	85 (80–89)	0.035 *	78 (76–81)	81 (75–92)	0.275
ALT (U/L)	25 (19–34)	25 (19–48)	0.079	30 (26–35)	27 (23–34)	0.735
AST (U/L)	23 (22–28)	23 (21–33)	0.255	28 (25–30)	25 (22–28)	0.572
GGT (U/L)	39 (26–58)	48 (25–73)	0.231	31 (27–49)	36 (30–48)	0.420
GLU (mmol/L)	5.3 (5.1–5.6)	5.3 (4.9–5.8)	0.579	5.2 (5.1–6.6)	5.1 (5.0–6.8)	0.243
TG (mmol/L)	1.57 (0.84–2.38)	1.41 (1.14–2.09)	0.484	1.92 (1.23–2.56)	1.73 (1.11–2.54)	0.599
TC (mmol/L)	4.20 (3.89–4.58)	4.13 (3.67–4.70)	0.833	4.44 (4.30–4.80)	4.38 (4.19–4.67)	0.290
GLO (g/L)	26 (24–29)	26 (23–28)	0.931	25 (23–27)	26 (23–29)	0.948

Data are shown as median (interquartile range). The asterisk represents the presence of significance. WBC, white blood cell count; HGB, hemoglobin; PLT, platelet count; CRE, creatinine; ALT, alanine aminotransferase; AST, aspartate aminotransferase; GGT, γ-glutamyl transpeptidase; GLU, blood glucose; TG, triglyceride; TC, total cholesterol; GLO, globulin.

## Data Availability

All data generated by the study are included in the manuscript or in its Supplementary Material.

## Supplementary Materials

The following supporting information can be downloaded at: https://www.mdpi.com/article/10.3390/v15122427/s1, Figure S1: Gating strategies used in this study.

## References

[1] Spinelli M.A., Jones B.L.H., Gandhi M. (2022). COVID-19 Outcomes and Risk Factors Among People Living with HIV. Curr. HIV/AIDS Rep..

[2] Ambrosioni J., Blanco J.L., Reyes-Uruena J.M., Davies M.A., Sued O., Marcos M.A., Martinez E., Bertagnolio S., Alcami J., Miro J.M. (2021). Overview of SARS-CoV-2 infection in adults living with HIV. Lancet HIV.

[3] Spinelli M.A., Lynch K.L., Yun C., Glidden D.V., Peluso M.J., Henrich T.J., Gandhi M., Brown L.B. (2021). SARS-CoV-2 seroprevalence, and IgG concentration and pseudovirus neutralising antibody titres after infection, compared by HIV status: A matched case-control observational study. Lancet HIV.

[4] Tebas P., Frank I., Lewis M., Quinn J., Zifchak L., Thomas A., Kenney T., Kappes R., Wagner W., Maffei K. (2010). Poor immunogenicity of the H1N1 2009 vaccine in well controlled HIV-infected individuals. AIDS.

[5] Johnston J.A., Tincher L.B., Lowe D.K. (2013). Booster and Higher Antigen Doses of Inactivated Influenza Vaccine in HIV-Infected Patients. Ann. Pharmacother..

[6] Antinori A., Cicalini S., Meschi S., Bordoni V., Lorenzini P., Vergori A., Lanini S., De Pascale L., Matusali G., Mariotti D. (2022). Humoral and Cellular Immune Response Elicited by mRNA Vaccination Against Severe Acute Respiratory Syndrome Coronavirus 2 (SARS-CoV-2) in People Living with Human Immunodeficiency Virus Receiving Antiretroviral Therapy Based on Current CD4 T-Lymphocyte Count. Clin. Infect. Dis..

[7] Feng Y., Zhang Y., He Z., Huang H., Tian X., Wang G., Chen D., Ren Y., Jia L., Wang W. (2022). Immunogenicity of an inactivated SARS-CoV-2 vaccine in people living with HIV-1: A non-randomized cohort study. eClinicalMedicine.

[8] Su B., Vanham G. (2023). Effectiveness of COVID-19 primary and booster vaccination in HIV-infected individuals. AIDS.

[9] Kim A.H.J., Nakamura M.C. (2022). COVID-19 Breakthrough Infection Among Immunocompromised Persons. JAMA Intern. Med..

[10] Sun J., Zheng Q., Madhira V., Olex A.L., Anzalone A.J., Vinson A., Singh J.A., French E., Abraham A.G., Mathew J. (2022). Association Between Immune Dysfunction and COVID-19 Breakthrough Infection After SARS-CoV-2 Vaccination in the US. JAMA Intern. Med..

[11] Lang R., Humes E., Coburn S.B., Horberg M.A., Fathi L.F., Watson E., Jefferson C.R., Park L.S., Gordon K.S., Akgün K.M. (2022). Analysis of Severe Illness After Postvaccination COVID-19 Breakthrough Among Adults with and without HIV in the US. JAMA Netw. Open.

[12] Yek C., Gianella S., Plana M., Castro P., Scheffler K., García F., Massanella M., Smith D.M. (2016). Standard vaccines increase HIV-1 transcription during antiretroviral therapy. AIDS.

[13] Fusco F.M., Carleo M.A., Sangiovanni N., D’Abbraccio M., Tambaro O., Borrelli F., Viglietti R., Camaioni C., Bruner V., Falanga R. (2023). Does COVID-19 Vaccination with BNT162b2 Influence HIV-Related Immunological and Virological Markers? Data from 235 Persons Living with HIV at Cotugno Hospital, Naples, Italy: Immune Response After Second and Third Doses, and Influence on Immunovirological Markers. Viral Immunol..

[14] Rai M.A., Shi V., Kennedy B.D., Justement J.S., Gittens K., McCormack G., Blazkova J., Moir S., Chun T.W. (2022). Effect of Severe Acute Respiratory Syndrome Coronavirus 2 Vaccine Booster on Human Immunodeficiency Virus Reservoirs and Im-mune Markers. Open Forum Infect. Dis..

[15] Günthard H.F., Wong J.K., Spina C.A., Ignacio C., Kwok S., Christopherson C., Hwang J., Haubrich R., Havlir D., Richman D.D. (2000). Effect of Influenza Vaccination on Viral Replication and Immune Response in Persons Infected with Human Immunodeficiency Virus Receiving Potent Antiretroviral Therapy. J. Infect. Dis..

[16] Gatechompol S., Avihingsanon A., Putcharoen O., Ruxrungtham K., Kuritzkes D.R. (2021). COVID-19 and HIV infection co-pandemics and their impact: A review of the literature. AIDS Res. Ther..

[17] Haidar G., Agha M., Bilderback A., Lukanski A., Linstrum K., Troyan R., Rothenberger S., McMahon D.K., Crandall M.D., Sobolewksi M.D. (2022). Prospective Evaluation of Coronavirus Disease 2019 (COVID-19) Vaccine Responses Across a Broad Spectrum of Immunocompromising Conditions: The COVID-19 Vaccination in the Immunocompromised Study (COVICS). Clin. Infect. Dis..

[18] Di Girolamo L., Ferrara M., Trevisan G., Longo B.M., Allice T., Burdino E., Alladio F., Fantino S., Di Perri G., Calcagno A. (2023). Transient plasma viral rebound after SARS-CoV-2 vaccination in an exceptional HIV-1 elite controller woman. Virol. J..

[19] Gong C., Song X., Li X., Lu L., Li T. (2021). Immunological changes after COVID-19 vaccination in an HIV-positive patient. Int. J. Infect. Dis..

[20] Lombardi A., Butta G.M., Donnici L., Bozzi G., Oggioni M., Bono P., Matera M., Consonni D., Ludovisi S., Muscatello A. (2021). Anti-spike antibodies and neutralising antibody activity in people living with HIV vaccinated with COVID-19 mRNA-1273 vaccine: A prospective single-centre cohort study. Lancet Reg. Health Eur..

[21] Vergori A., Lepri A.C., Cicalini S., Matusali G., Bordoni V., Lanini S., Meschi S., Iannazzo R., Mazzotta V., Colavita F. (2022). Immunogenicity to COVID-19 mRNA vaccine third dose in people living with HIV. Nat. Commun..

[22] Levy I., Wieder-Finesod A., Litchevsky V., Biber A., Indenbaum V., Olmer L., Huppert A., Mor O., Goldstein M., Levin E.G. (2021). Immunogenicity and safety of the BNT162b2 mRNA COVID-19 vaccine in people living with HIV-1. Clin. Microbiol. Infect..

[23] Heftdal L.D., Perez-Alos L., Hasselbalch R.B., Hansen C.B., Hamm S.R., Moller D.L., Pries-Heje M., Fogh K., Gerstoft J., Gronbaek K. (2023). Humoral and cellular immune responses eleven months after the third dose of BNT162b2 an mRNA-based COVID-19 vaccine in people with HIV—A prospective observational cohort study. eBioMedicine.

[24] Matveev V.A., Mihelic E.Z., Benko E., Budylowski P., Grocott S., Lee T., Korosec C.S., Colwill K., Stephenson H., Law R. (2023). Immunogenicity of COVID-19 vaccines and their effect on HIV reservoir in older people with HIV. iScience.

[25] Lv X., Zhao C., Song B., Huang H., Song S., Long H., Liu W., Du M., Liu M., Liu J. (2023). COVID-19 vaccination in people living with HIV and AIDS (PLWHA) in China: A cross-sectional study. Hum. Vaccines Immunother..

[26] Steenberg B., Sokani A., Myburgh N., Mutevedzi P., Madhi S.A. (2023). COVID-19 Vaccination Rollout: Aspects of Hesitancy in South Africa. Vaccines.

[27] Calza L., Bon I., Borderi M., Colangeli V., Viale P. (2020). No Significant Effect of COVID-19 on Immunological and Virological Parameters in Patients with HIV-1 Infection. J. Acquir. Immune Defic. Syndr..

[28] Hu R., Yan H., Liu M., Tang L., Kong W., Zhu Z., Liu P., Bai W., Hu X., Ding J. (2020). Brief Report: Virologic and Immunologic Outcomes for HIV Patients with Coronavirus Disease 2019. Am. J. Ther..

[29] Pasternak A.O., Lukashov V.V., Berkhout B. (2013). Cell-associated HIV RNA: A dynamic biomarker of viral persistence. Retrovirology.

[30] Yukl S.A., Shergill A.K., Ho T., Killian M., Girling V., Epling L., Li P., Wong L.K., Crouch P., Deeks S.G. (2013). The Distribution of HIV DNA and RNA in Cell Subsets Differs in Gut and Blood of HIV-Positive Patients on ART: Implications for Viral Persistence. J. Infect. Dis..

[31] Sung J.M., Margolis D.M. (2018). HIV Persistence on Antiretroviral Therapy and Barriers to a Cure. Adv. Exp. Med. Biol..

[32] Wherry E.J., Barouch D.H. (2022). T cell immunity to COVID-19 vaccines. Science.

[33] Koutsakos M., Reynaldi A., Lee W.S., Nguyen J., Amarasena T., Taiaroa G., Kinsella P., Liew K.C., Tran T., Kent H.E. (2023). SARS-CoV-2 breakthrough infection induces rapid memory and de novo T cell responses. Immunity.

[34] Hyun H., Jang A.-Y., Park H., Heo J.Y., Bin Seo Y., Nham E., Yoon J.G., Seong H., Noh J.Y., Cheong H.J. (2023). Humoral and cellular immunogenicity of homologous and heterologous booster vaccination in Ad26.COV2.S-primed individuals: Comparison by breakthrough infection. Front. Immunol..

[35] Fu J.Y.L., Pukhari M.H., Bador M.K., Sam I.C., Chan Y.F. (2023). Humoral and T Cell Immune Responses against SARS-CoV-2 after Primary and Homologous or Heterologous Booster Vaccinations and Breakthrough Infection: A Longitudinal Cohort Study in Malaysia. Viruses.

[36] Zhang J., He Q., An C., Mao Q., Gao F., Bian L., Wu X., Wang Q., Liu P., Song L. (2021). Boosting with heterologous vaccines effectively improves protective immune responses of the inactivated SARS-CoV-2 vaccine. Emerg. Microbes Infect..

[37] Yi X., Wang Y., Li Q., Li X., Zhang P., Fu X., Gu S., Zhang D., Liu X., Lou H. (2023). Pre-existing immunity to SARS-CoV-2 associates with strong T cell responses induced by inactivated COVID-19 vaccines. J. Med. Virol..

