# Supplementary material for: Effect of SARS-CoV-2 Breakthrough Infection on HIV Reservoirs and T-Cell Immune Recovery in 3-Dose Vaccinated People Living with HIV
Source: Viruses. 2023 Dec 14;15(12):2427. doi: 10.3390/v15122427 (PMC10748120; doi:10.3390/v15122427)
Supplement: Supplementary file 1 [file viruses-15-02427-s001.zip › viruses-2726371-supplementary.pdf]

Supplementary Materials

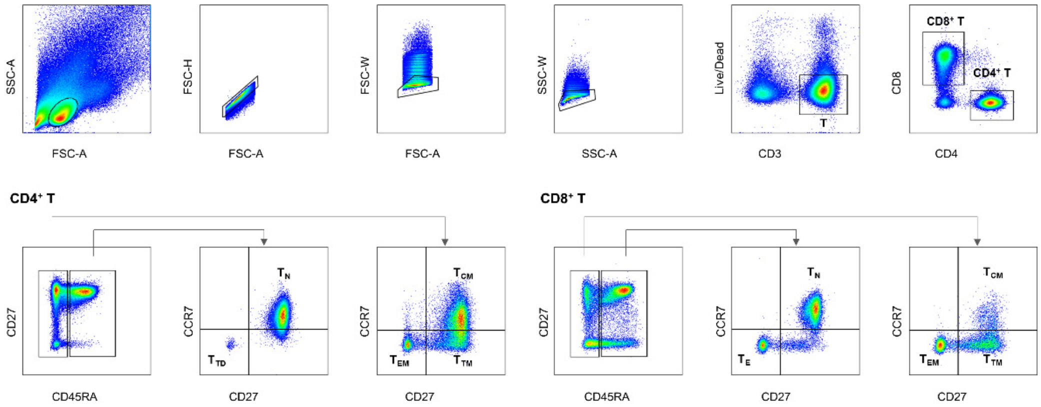

Supplementary Figure S1. Gating strategies used in this study. T cells were identified by gating on lymphocytes (FSC-A vs. SSC-A), singlets (FSC-A vs. FSC-H, FSC-A vs. FSC-W, and SSC-A vs. SSC-W) and live T cells (CD3 vs. Live/Dead). CD4 and CD8 T cells were identified using anti-CD4 and anti-CD8 antibodies, respectively. CD4 and CD8 T cell subsets were further identified b CD45RA, CD27, and CCR7 expression: naïve (T_{N}, CD45RA^{+}CD27^{+}CCR7^{+}), central memory (T_{CM}, CD45RA^{-}CD27^{+}CCR7^{-}), transitional memory (T_{TM}, CD45RA^{-}CD27^{+}CCR7^{-}), effector memory (T_{EM}; CD45RA^{-}CD27^{-}CCR7^{-}), and terminal differentiated/effector (T_{TD}/T_{E}, CD45RA^{+}CD27^{+}CCR7^{-}).
